# Identification of CD4 and H-2K^d^-restricted cytotoxic T lymphocyte epitopes on the human herpesvirus 6B glycoprotein Q1 protein

**DOI:** 10.1038/s41598-019-40372-5

**Published:** 2019-03-07

**Authors:** Satoshi Nagamata, Taiki Aoshi, Akiko Kawabata, Yoshiaki Yamagishi, Mitsuhiro Nishimura, Soichiro Kuwabara, Kouki Murakami, Hideto Yamada, Yasuko Mori

**Affiliations:** 1 Division of Clinical Virology, Center for Infectious Diseases, Kobe University Graduate School of Medicine, Kobe, Hyogo, Japan; 2 Vaccine Dynamics Project, BIKEN Innovative Vaccine Research Alliance Laboratories, Research Institute for Microbial Disease, Osaka University, Osaka, Japan; 3 Kanonji Institute, Seto Center, The Research Foundation for Microbial Diseases of Osaka University, Kanonji-shi, Kagawa, Japan; 4 Department of Obstetrics and Gynecology, Kobe University Graduate School of Medicine, Kobe, Hyogo, Japan

## Abstract

The identification of Human herpesvirus 6B (HHV-6B) epitopes that are recognized by T-cells could contribute to the development of potential vaccines and immunotherapies. Here, we identified CD4^+^ and H-2K^d^-restricted CD8^+^ T-cell epitopes on the glycoprotein Q1 of HHV-6B (BgQ1), which is a unique glycoprotein and essential for HHV-6B viral entry, by using *in vivo* electroporation with a plasmid DNA encoding BgQ1, overlapping peptides spanning the BgQ1 sequence, ELISPOT assay for quantification of gamma interferon (IFN-γ), and computer-based T-cell epitope prediction programs. The CD4^+^ and CD8^+^ T-cell epitopes identified in BALB/c mice in this study could be a good animal model system for use in the development of T-cell responses, inducing HHV-6B vaccines or immunotherapies.

## Introduction

Human herpesvirus 6 (HHV-6), which belongs to the betaherpesvirus subfamily, was first isolated from patients with lymphocytic disorders in 1986^1^. Since 2012, HHV-6 has been classified as two independent virus species, HHV-6A and HHV-6B^2^, based on genetic and antigenic differences and cell tropism^3–5^. HHV-6B is the causative agent for exanthem subitum^6^ and is sometimes associated with severe encephalopathy, while the pathogenesis of HHV-6A is still unknown. More than 90% of individuals are infected with HHV-6B during childhood, and the virus remains latent after primary infection^7,8^. HHV-6B reactivation causes life-threatening encephalitis in immunosuppressed patients^9^ and is also associated with drug-induced hypersensitivity syndrome^10^. However, effective immunotherapies based on antibodies, expanded T-cells, or vaccines for controlling HHV-6B infection and reactivation have not been established.

Glycoproteins or their complexes on the surface of enveloped viruses play pivotal roles in the viruses’ infectivity. Glycoprotein Q1 (gQ1) and gQ2 are unique genes that are encoded specifically in HHV-6 and human herpesvirus 7 (HHV-7). Recently, our group reported that human CD134, also called OX40, is a specific cellular receptor for HHV-6B and binds to the HHV-6B gH/gL/gQ1/gQ2 complex^11^. Moreover, the HHV-6B gQ1 (BgQ1) and gQ2 (BgQ2) subunits are sufficient for CD134 binding, and a region in BgQ1 is critical for the function of the HHV-6B gH/gL/gQ1/gQ2 complex^12^. When screening for monoclonal antibodies (MAbs) with neutralizing activity against HHV-6B, the neutralizing MAbs obtained were almost all against BgQ1^13^. Thus, the BgQ1 protein, which is unique to HHV-6B, seems to be critical for virus entry and adaptive immunity.

The recognition by CD8^+^ cytotoxic T lymphocytes (CTL) of antigen peptides presented by class I human leucocyte antigen (HLA) is an essential step in adaptive immunity for virus infection. Identifying immunodominant proteins and epitopes could lead to the design of potential new vaccines and immunotherapies. Recently, some studies have identified HHV-6 antigens that are targeted by the CD4^+^ and CD8^+^ T-cell responses^14,15^. Most of these focused on functional or positional homologs to known T-cell antigens from human cytomegalovirus (HCMV) in chronically infected adults, because HCMV also belongs to the same betaherpesvirus subfamily as HHV-6B. The T-cells responding to the identified HHV-6 antigens are present at low frequency in healthy adults and need to be expanded *in vitro* for use in autologous immunotherapy^16–18^. In addition, to design vaccines and immunotherapies for HHV-6B infection in humans, a good animal model will be needed to analyze T-cell responses against the HHV-6B antigen.

DNA vaccines can engender not only humoral but also cellular immune responses^19^. And they offer some advantages including easy construction, preparation, stability. The vaccine immunogenicity and efficacy are significantly enhanced by *in vivo* electroporation^20^.

In this report, we attempted to determine T-cell epitopes on BgQ1, which is a unique glycoprotein and essential for HHV-6B viral entry. Due to the similarities in the peptide-binding motifs between H-2K^d^ and HLA-A24, BALB/c mice were chosen as the animal model hosts^21–24^. We identified an H-2K^d^-restricted CD8^+^ T-cell epitope in BALB/c mice by using *in vivo* electroporation with a plasmid DNA encoding BgQ1, overlapping peptides spanning the BgQ1 sequence, ELISPOT assay for quantification of gamma interferon (IFN-γ), and computer-based T-cell epitope prediction programs.

## Results

### Identification of CD4^+^ or CD8^+^ T-cell stimulating peptides by using the synthetic overlapping peptides from BgQ1

**Figure 1:**
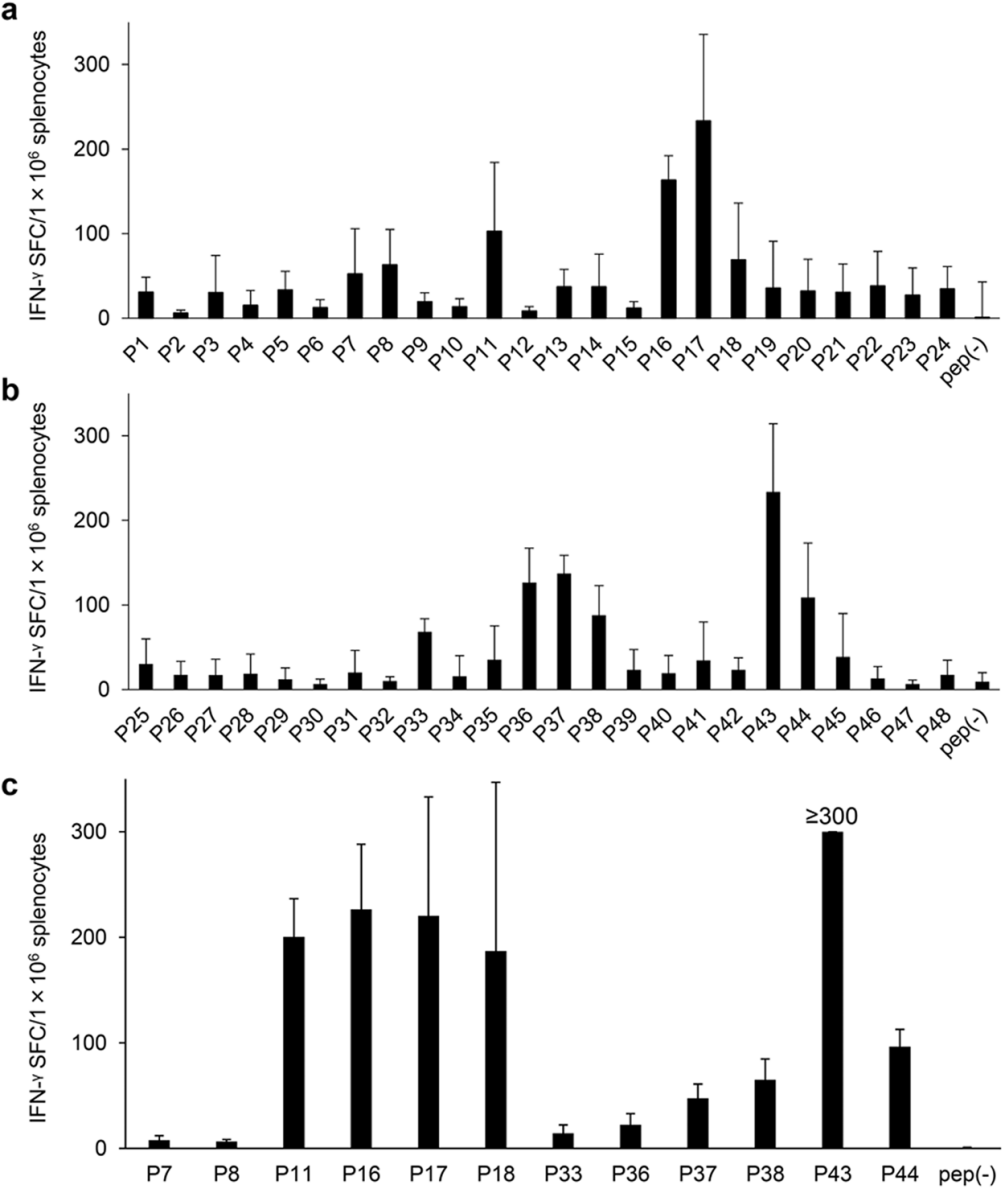
IFN-γ ELISPOT assay of splenocytes from BALB/c mice immunized with pCAGGS-MCS BgQ1m. Overlapping peptides (5 µg/ml for each peptide) from glycoprotein Q1 of human herpesvirus 6B (BgQ1) were used for stimulation. Peptides were divided into two groups: the first group contained the first 24 peptides (P1-P24) (**a**) and the second group contained the other 24 peptides (P25-P48) (**b**). (**c**) Splenocytes from immunized mice were stimulated with the selected 12 peptides (5 µg/ml for each peptide). The results were expressed as spot forming cells (SFC)/1 × 10^6^ splenocytes and the wells of “not reliably countable high signals” were defined as 300 SFC/1 × 10^6^ splenocytes. Pep(-) indicates medium alone. The data are the mean ± SD of duplicate (**a**,**b**) and triplicated (**c**) wells. And the data are representative of three independent experiments. Independent experiments were carried out from independent mice.

As a first preliminary ELISPOT assay, overlapping peptides from BgQ1 (P1–P48) were used for stimulation. Peptides were divided into two groups: the first group contained the first 24 peptides (P1–P24) and the second group contained the other 24 peptides (P25–P48). Splenocytes from BALB/c mice immunized with a plasmid DNA encoding BgQ1 were stimulated with the individual peptides for 40 h, and IFN-γ spots were counted. Independent experiments were carried out from independent mice. As shown in Fig. 1a,b, splenocytes in the presence of 12 peptides (P7, P8, P11, P16, P17, P18 from the first half, and P33, P36, P37, P38, P43, P44 from the second half) showed more than 50 SFC/1 × 10^6^ splenocytes.

**Figure 2:**
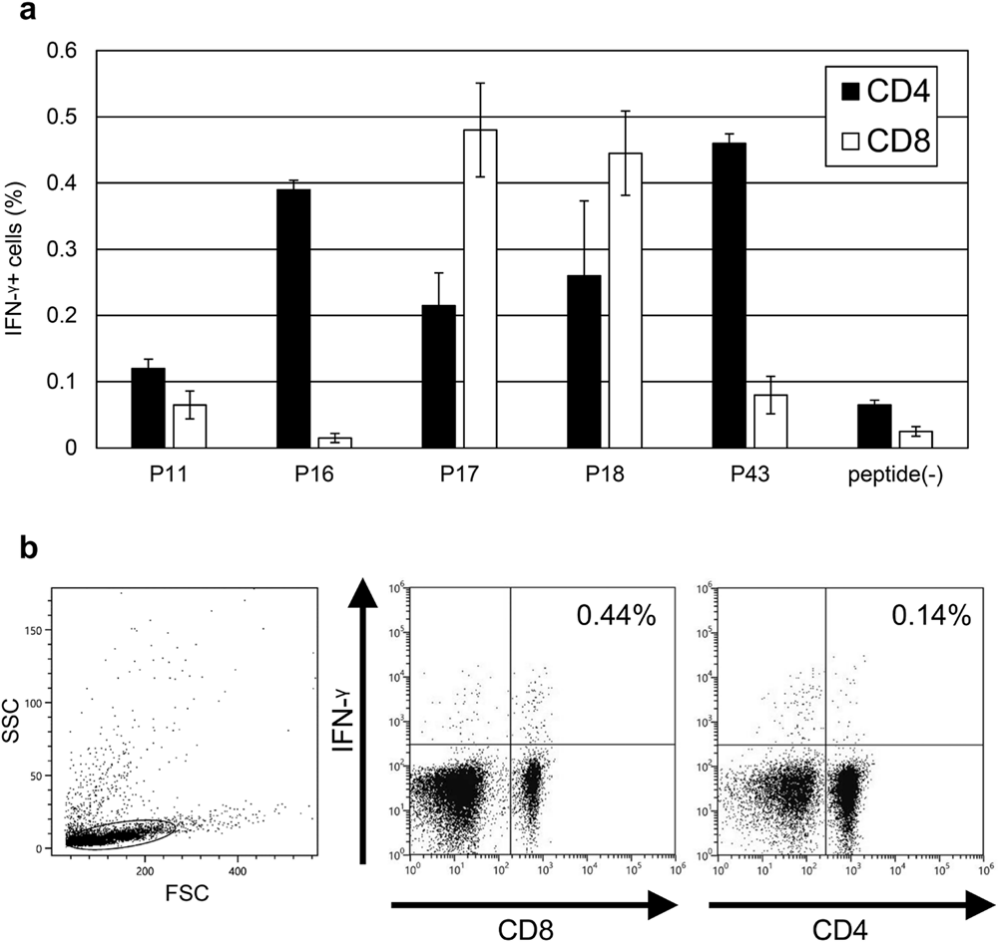
Identification of peptides inducing CD8^+^ T-cell responses. IFN-γ-producing T-cells in the spleens of BALB/c mice immunized with pCAGGS-MCS BgQ1m. (**a**) Three-color flow cytometric analysis was performed for the detection of intracellular IFN-γ and cell surface CD4 and CD8 molecules after immune splenocytes were cultured in the presence of the 5 candidate peptides. The data are the percentages of IFN-γ-producing CD4^+^ or CD8^+^ cells in lymphocyte after 4 h of stimulation with peptides (mean ± SD of duplicates). Peptide(-) indicates medium alone. The data are representative of three independent experiments with similar results. (**b**) A representative flow cytometry plot of intracellular IFN-γ and CD8 and CD4 in response to P17 peptide. The lymphocytes were gated by forward scatter (FSC) and side scatter (SSC) and then intracellular IFN-γ levels were detected on CD4^+^ and CD8^+^ cells in the lymphocytes. The data are shown as the percentages of IFN-γ-producing cells in CD4^+^ and CD8^+^ lymphocyte gate.

In a second preliminary ELISPOT assay, splenocytes from immunized mice were stimulated with the 12 peptides that showed more than 50 SFC/1 × 10^6^ splenocytes in the first ELISPOT. As shown in Fig. 1c, substantial IFN-γ production was confirmed in splenocytes after stimulation with 5 peptides (P11, P16, P17, P18, and P43). The remaining 7 peptides were considered to have lower immunogenicity. These preliminary results were further confirmed by an independently performed three-color flow cytometric analysis. To reveal the responsive T-cell subset, the findings of intracellular IFN-γ staining after stimulation with these 5 peptides were examined. The results demonstrated that CD8^+^ T-cells mainly produced IFN-γ in response to P17 and P18 (Fig. 2). On the other hand, CD4^+^ T-cells mainly produced IFN-γ in the presence of P16 and P43. CD4^+^ T-cells also produced IFN-γ in the presence of P11, but the IFN-γ signals were lower than the other 4 peptides.

### Identification of an MHC class Ia restriction molecule for P17 and P18 of BgQ1

**Figure 3:**
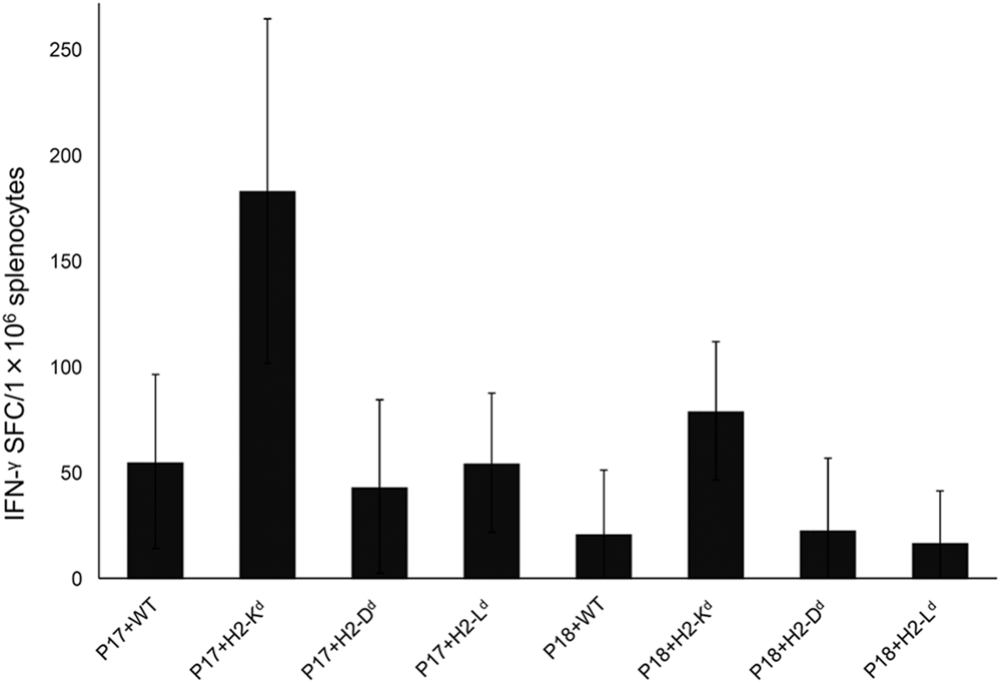
Identification of MHC class Ia restriction molecules for P17 and P18 of BgQ1. BW5147 (H2k) lymphoma cell lines expressing either H-2K^d^, H-2D^d^, H-2L^d^, or just the wild type were co-cultured with P17 or P18, and used for stimulation of splenocytes from BALB/c mice immunized with pCAGGS-MCS BgQ1m. IFN-γ productions were measured by ELISPOT assay. Results are expressed as spot forming cells (SFC)/1 × 10^6^ splenocytes. The data are the mean ± SD of three independent experiments of one mouse.

Since P17 and P18 were found to stimulate CD8^+^ T-cells, we next tried to determine which MHC class Ia molecule was involved in the presentation of peptides to CD8^+^ T-cells. BW5147 (H2k) lymphoma cell lines expressing either H-2K^d^, H-2D^d^, H-2L^d^, or just the wild type molecule (H2k) were co-cultured with P17 or P18, and used for stimulation of splenocytes from BgQ1-immunized mice. IFN-γ production for both P17 and P18 was only observed in H-2K^d^ expressing BW5147 (Fig. 3), indicating P17 and P18 contained the H-2K^d-^restricted CD8^+^ T-cell epitope.

### Identification of a 9-mer CD8^+^ T-cell epitope on P17 and P18 of BgQ1

Several CD8^+^ T-cell epitope candidates within the P17 and P18 were predicted by using two computer-based programs BIMAS HLA Peptide Binding Prediction (http://bimas.dcrt.nih.gov/cgi-bin/molbio/ken_parker_comboform) and SYFPEITHI Epitope Prediction (http://www.syfpeithi.de/). Table 1 shows the results of these programs. Since P17 and P18 are both presented by H-2K^d^, 4 peptides which had high scores were synthesized as epitope candidates: an 8-mer (FCPMTSKL), a 9-mer (AFCPMTSKL), and a 10-mer (IAFCPMTSKL) peptide from the overlapping region of P17 and P18; and a 9-mer peptide (KPLTAMTAI) from the front half of P17.

**Table 1:** Candidate T-cell epitopes on the P17 and P18 peptide of the BgQ1m molecule.

Peptide	Amino acid sequence^a^	Estimated scores for restriction molecules^b^					
		BIMAS			SYFPEITHI		
		D^d^	K^d^	L^d^	D^d^	K^d^	L^d^
P17	RLKPLTAMTAIAFCPMTSKL						
	AFCPMTSKL	1	1382.4	—	—	20	12
	KPLTAMTAI	0.6	115.2	30	—	22	18
	FCPMTSKL	24	48	—	—	—	—
	IAFCPMTSKL	1	48	5	—	12	—
	KPLTAMTAIA	0.12	2.**88**	30	—	11	—
P18	IAFCPMTSKLELRQNYRLEK						
	AFCPMTSKL	1	1382.4	5	—	20	12
	CPMTSKLEL	0.3	115.2	150	—	17	22
	FCPMTSKL	7.2	57.6	7.5	—	11	—
	IAFCPMTSKL	1	48	5	—	12	—
	KLELRQNYRL	1.2	48	1.5	—	12	—

^a^Boldface type indicates peptide sequences which were synthesized and used for experiments. Underlining indicates anchor residues of K^d^-restricted T-cell nonamer epitopes.

^b^no binding score.

**Figure 4:**
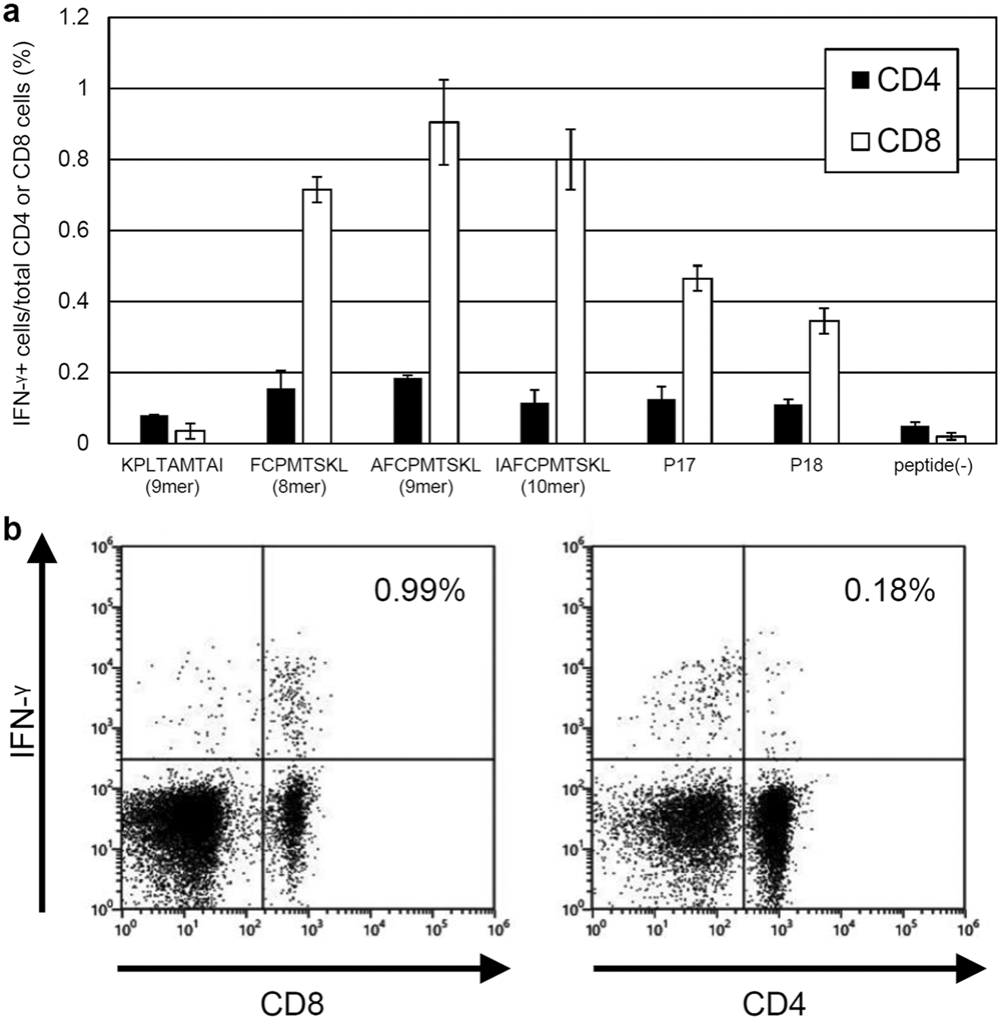
Determination of an optimal T-cell epitope on the P17 and P18 peptides and a T-cell subset recognizing the epitope in BALB/c mice. (**a**) IFN-γ-producing T-cell subsets in the spleens of BALB/c mice immunized with pCAGGS-MCS BgQ1m. Three-color immunofluorescence analysis was performed on flow cytometer to detect intracellular IFN-γ, cell surface CD4, and CD8 molecules after immune splenocytes were cultured in the presence of the peptides. The data are the percentages of IFN-γ-producing CD4^+^ or CD8^+^ cells in lymphocyte after 4 h of stimulation with peptides (mean ± SD of duplicates). Peptide(-) indicates medium alone. Representative data from three independent experiments with similar results are shown. (**b**) A representative flow cytometry plot of intracellular IFN-γ and CD8 (left) and CD4 (right) in response to the 9-mer peptide (AFCPMTSKL) from an overlapping region of P17 and P18. Very few intracellular IFN-γ-positive CD4^+^ T-cells were observed after stimulation with the AFCPMTSKL peptide (right). The data are shown as the percentages of IFN-γ-producing cells in lymphocyte gate.

As shown in Fig. 4a,b, the 9-mer peptide (AFCPMTSKL) from the overlapping region of P17 and P18 induced the strongest intracellular IFN-γ signals in the CD8^+^ T-cells by flow cytometric analysis, followed by the 10-mer (IAFCPMTSKL) and 8-mer (FCPMTSKL) peptides, indicating that this 9mer peptide is the optimal H-2K^d^-restricted CD8^+^ T-cell epitope on BgQ1, in BALB/c mice. Another 9-mer peptide (KPLTAMTAI) from the front half of P17 induced only a background level of intracellular IFN-γ synthesis.

## Discussion

In this study, we identified an H-2K^d^-restricted CD8^+^ T-cell epitope on the BgQ1 molecule. In addition, we found that P43 and P16 of the BgQ1 contain CD4^+^ T-cell epitopes in BALB/c mice.

In general, CTL plays a crucial role in protective immunity against infection with intracellular pathogens, such as certain bacteria and viruses^25,26^. Although many things are still unknown about the mechanisms of T-cell mediated protection against HHV-6B infection, the necessity of T-cells to control HHV-6B replication is suggested by the higher incidence of persistent HHV-6B viremia in patients without proliferative T-cell responses^27^. To better understand CD8^+^ T-cell responses against HHV-6B infection, identifying the epitopes which are recognized by CD8^+^ T-cells is important. However, the HHV-6B genome encodes hundreds of proteins, making the identification of immunodominant epitopes complicated. Recent reports which defined CD4^+^ and CD8^+^ T-cell epitopes by using expanded T-cells *in vitro* focused on the HHV-6 proteins present at high levels in virus preparations^18^, or on the HHV-6 homologies of antigens defined for HCMV^16,17^. In this study, we focused on the BgQ1 protein, which plays an essential role in HHV-6B virus entry and is a promising candidate for antiviral therapy^13^.

In the present mouse model, we identified the H-2K^d^-restricted CTL epitope on the BgQ1 molecule. This epitope is not conserved in HHV-6A gQ1 sequence (strain U1102; PubMed accession NC 001664), but similar sequence (RFCPMTTKL) which has same amino acids in the main anchor positions of nonameric K^d^ epitope is present (position 2 and 9).

In this study, mice were immunized with a codon-optimized plasmid DNA expressing the BgQ1 by *in vivo* electroporation to identify the CTL epitopes. DNA vaccination is a powerful tool for identifying T-cell epitopes. However, optimization of codon usage is an important consideration in constructing DNA vaccines^26^. DNA immunization with an optimized codon has been reported to result in enhanced CTL reactivity by increasing the translational efficiency of plasmid DNA^28,29^. Moreover, an *in vivo* electroporation technique to induce transient and reversible permeabilization of the cell membrane improves the efficiency of plasmid DNA transfection^30^. Electroporation enhances the immunogenicity and effectiveness of DNA vaccines by increasing antigen delivery up to 1000-fold over naked DNA delivery alone^20^.

IFN-γ producing cells were detected by three-color flow cytometric analysis. It might have been better if we had used Live/Dead and other markers of CTL activity, because the percentages of IFN-γ-producing cells in this study were relatively small. These markers may influence our results. However, similar number of IFN-γ producing cells with similar system were reported in another study^31,32^ and IFN-γ production was consistently detected over the background level.

We also used the computer-based algorithm programs BIMAS and SYFPEITHI to predict the epitopes^33–35^. The strategy including DNA vaccines, overlapping peptide and these computer-based programs is an effective methods for narrowing down the amino acid region of the T-cell epitope^31^. Prediction in these programs is restricted to for 8–11mer length peptides. It would be possible CTL can recognize much longer peptides up to 14mer^36^, however we did not pursue this long CTL peptide in this study. We also found that P43 and P16 of the BgQ1 contain CD4^+^ T-cell epitopes in BALB/c mice. Especially, P43 contains 15mer peptide which showed high score (VNNIFTVQARYSKQN, score 31) for binding to the H-2A^d^ molecule by SYFPEITHI Epitope Prediction. But MHC class II prediction tools do not perform as well as class I predictions^37^, there is room for further research.

In conclusion, we identified an H-2K^d^-restricted CD8^+^ T-cell epitope on the BgQ1 molecule and two peptides which contain CD4^+^ T-cell epitopes in BALB/c mice. These results warrant further study to examine whether the epitopes are applicable to animal models or humans and could be utilized in vaccines or immunotherapies.

## Materials and Methods

### Mice

Six-week-old female BALB/c mice (16–20 g) were obtained from Japan SLC (Shizuoka, Japan). The mice were housed in-house under specific pathogen-free conditions maintained at 22 ± 2 °C and 55 ± 5% relative humidity, in a 12-hour light/dark cycle environment, and provided with food and water *ad libitum*. The health condition of the mice was monitored daily. A total of 25 mice were used including preliminary experiments. All mice were immunized with plasmid DNA and each experiment was independently repeated three times. All of the animal experimental procedures were approved by the Kobe University Institutional Animal Care and Use Committee (Permission number: P131101-R1) and carried out according to the Animal Experimentation Regulations of Kobe University.

### Construction of the plasmid DNAs, pCAAGGS-MCS BgQ1m

To optimize the gene expression in mammalian cells, the BgQ1 original cDNA (GenBank accession no. MK388090) codon was converted to more common in human genes and cloned into the pMX plasmid by Invitrogen (Carlsbad, CA). The amplified BgQ1 DNA fragments were digested with SacI and KpnI and ligated into the digested pCAGGS-MCS vector, yielding the plasmid pCAGGS-MCS BgQ1m. The plasmid was amplified in DH5α *Escherichia coli* and purified using QIAGEN Plasmid Mega Kit (QIAGEN, Hilden, Germany) following the manufacturer’s instructions.

### Peptides

BgQ1 of the HHV-6B HST strain has 516-amino-acid (aa). Except for the signal sequence (aa 1 to 25), peptides spanning the total 491 aa BgQ1 sequence were prepared as 20-mers overlapping by 10 residues (Supplementary Fig. S1). Forty-eight lyophilized peptides in total (P1–P48) were purchased from Eurofins Genomics (Tokyo, Japan). The purity of the peptides was 50% or more, confirmed by high-performance liquid chromatography (HPLC) and verified for correct sequence by mass spectroscopy (MS). All peptides were dissolved in dimethyl sulfoxide (DMSO) at a concentration of 10 mg/ml and stored at −80 °C until use.

### Immunization of mice

Mice were immunized with a plasmid DNA expressing the BgQ1 by *in vivo* electroporation. After anesthesia by isoflurane, 100 µg of pCAGGS-MCS BgQ1m was injected twice into both femoral muscles of the mice, and an NEPA21 electroporator (NepaGene, Tokyo, Japan) was used for transfection. Immunizations were performed three times at 2-week intervals and the spleens were removed one week after the third immunization.

### Cell lines

BW5147 (H2k) lymphoma cell line (JCRB9002) was obtained from JCRB Cell Bank (Japan). BW5147 cells retrovirally transduced with one of the genes encoding H-2K^d^, H-2D^d^, or H-2L^d^ were made as previously described^32^, and used to determine the CD8^+^ T-cell epitope-presenting MHC Ia molecule. The cells were cultured in RPMI 1640 medium with 10% heat-inactivated fetal bovine serum (RPMI/10FBS) in an incubator with a humidified atmosphere containing 5% CO_2_. FBS, which originate from Canada (endotoxin level ≤ 50 EU/mL, hemoglobin level ≤ 25 mg/dL), was purchased from Gibco (cat. # 12483020, Thermo Fisher Scientific, Waltham, MA).

### Preparation of splenocytes from an immunized mouse

Immunized mice were euthanized by an intraperitoneal injection of sodium pentobarbital and cervical dislocation. The spleens were aseptically removed and single cell suspensions were prepared after dissociation of the spleen through a cell strainer. Splenocytes were isolated by Ficoll density gradient centrifugation, suspended in RPMI/10FBS at a concentration of 1 × 10^7^ cells/ml, and used for assay.

### Quantification of IFN-γ by ELISPOT assay

The wells of a 96-well MultiScreen HA plate (Millipore, Billerica, MA) were precoated and blocked with RPMI/10FBS according to the manufacturer’s instructions (Ready-Set-Go Mouse IFN-γ ELISPOT kit; eBioscience, San Diego, CA). After blocking, 50 µl of RPMI/10FBS was added to each well followed by 100 µl of splenocytes (1 × 10^6^ cells) from immunized mice. All dissolved peptides in DMSO were diluted in RPMI/10FBS to a concentration of 20 µg/ml, and 50 µl of the resulting solution was added to each well. The total volume was 200 µl/well and the final concentration of each peptide was 5 µg/ml. Measurements were made in duplicate for each peptide in the first preliminary ELISPOT, and in triplicate in the second preliminary ELISPOT.

The plates were incubated for 40 h at 37 °C in a 5% CO_2_ humidified incubator. IFN-γ spot-forming cells (SFC) were detected and developed according to the manufacturer’s instructions (Ready-Set-Go Mouse IFN-γ ELISPOT kit; eBioscience). As a minor modification, 100 µl of 3,3′,5,5′-tetramethylbenzidine-H substrate (Moss, Pasadena, MD) was used for development of spots, and the spots were developed for 3 min at room temperature. After stopping the substrate reaction by washing wells with water, the plates were dried. The spots were then enumerated using the KS ELISPOT system (Carl Zeiss Microscopy GmbH, Jena, Germany) for automated spot counting. Because too many spots can cause signal overlapping and a reduction in counting accuracy, the wells of undercounting high signals were defined as 300 SFC per 1 × 10^6^ splenocytes.

### Intracellular IFN-γ staining and flow cytometry

Splenocytes from the immunized mice were treated with ammonium chloride-tris (ACT) buffer for 3 min at room temperature to remove red blood cells. The cells were then washed twice with RPMI 1640 medium and resuspended in RPMI/10FBS at a concentration of 1 × 10^7^ cells/ml. The cells (2 × 10^6^ cells) were incubated for 4 h at 37 °C in the presence or absence of peptide at a concentration of 10 µg/ml with Golgistop stock solution (monensin solution; BD Biosciences, San Jose, CA) diluted 1:1,500. The cells were then washed twice with 2% bovine serum albumin (BSA) in phosphate buffered saline (PBS) followed by staining with fluorescein isothiocyanate (FITC)-conjugated anti-CD8 (clone KT15; MBL, Nagoya, Japan) and phycoerythrin-indotricarbocyanine (PE/Cy7)-conjugated anti-CD4 (clone GK1.5; BioLegend, San Diego, CA) monoclonal antibodies (mAbs) for 30 min at 4 °C. The cells were washed twice and then intracellular cytokine staining (ICS) was performed by using a BD Cytofix/Cytoperm kit (BD Biosciences) according to the manufacturer’s protocol. ICS for IFN-γ was performed with PE-conjugated anti-IFN-γ (clone XMG1.2; BD Biosciences) mAbs for 30 min at 4 °C. The cells were washed twice, resuspended in PBS with 2% BSA, and then analyzed by flow cytometry with a SA3800 spectral analyzer (Sony Corporation, Tokyo, Japan).

### Determination of the restricted MHC Ia molecule

The CD8^+^ T-cell epitope-presenting H-2^d^ molecules, i.e., H-2K^d^, H-2D^d^, and H-2L^d^, were identified as previously reported^32^. Briefly, BW5147-K^d^, BW5147-D^d^, BW5147-L^d^, or BW5147 wild type cells (4 × 10^6^ cells) were co-cultured with each peptide (10 µg/ml) at 37 °C for 1 h. The cells were washed three times with RPMI 1640 medium and resuspended in RPMI/10FBS at a concentration of 4 × 10^6^ cells/ml. Splenocytes (1 × 10^6^ cells) from the immunized mice were stimulated with 2 × 10^5^ of each type of peptide-pulsed BW5147 cells in 200 µl of RPMI/10FBS for 40 h at 37 °C, and the levels of IFN-γ production were determined by ELISPOT assay.

## Data Availability

The datasets generated during and/or analyzed during the current study are available from the corresponding author on reasonable request.

## Supplementary information

Figure S1
