# Supplementary material for: Identification of CD4 and H-2Kd-restricted cytotoxic T lymphocyte epitopes on the human herpesvirus 6B glycoprotein Q1 protein
Source: Sci Rep. 2019 Mar 7;9:3911. doi: 10.1038/s41598-019-40372-5 (PMC6405833; doi:10.1038/s41598-019-40372-5)
Supplement: Supplementary file 1 — Figure S1 [file 41598_2019_40372_MOESM1_ESM.docx]

**Identification of CD4 and H-2K^d^-restricted cytotoxic T lymphocyte epitopes on the human herpesvirus 6B glycoprotein Q1 protein**

Satoshi Nagamata^1, 4^, Taiki Aoshi^2^, Akiko Kawabata^1^, Yoshiaki Yamagishi^1^, Mitsuhiro Nishimura^1^, Soichiro Kuwabara^2,3^, Kouki Murakami^1, 3^ , Hideto Yamada^4^, Yasuko Mori^1^*

^1^Division of Clinical Virology, Center for Infectious Diseases, Kobe University Graduate School of Medicine, Kobe, Hyogo, Japan

^2^Vaccine Dynamics Project, BIKEN Innovative Vaccine Research Alliance Laboratories, Research Institute for Microbial Disease, Osaka University, Osaka, Japan

^3^Kanonji Institute, Seto Center, The Research Foundation for Microbial Diseases of Osaka University, Kanonji-shi, Kagawa, Japan

^4^Department of Obstetrics and Gynecology, Kobe University Graduate School of Medicine, Kobe, Hyogo, Japan

^*^Corresponding author

E-mail: [ymori@med.kobe-u.ac.jp](mailto:ymori@med.kobe-u.ac.jp)

**Supplementary information**

**Figure S1**. **Schematic representation of the 48 overlapping peptides from glycoprotein Q1 of human herpesvirus 6B (BgQ1).** Peptides spanning the entire 491-amino-acid (aa) BgQ1 sequence of the human herpesvirus 6B HST strain, except for the signal sequence (aa 1 to 25). Forty-eight lyophilized peptides in total (P1–P48) were prepared.
